# Editorial: Magnetic Resonance-guided Focused Ultrasound (MRgFUS)

**DOI:** 10.3389/fneur.2025.1658187

**Published:** 2025-08-12

**Authors:** Francesca Pistoia, Marc Gallay, Michele Tinazzi

**Affiliations:** ^1^Department of Biotechnological and Applied Clinical Sciences, University of L‘Aquila, L'Aquila, Italy; ^2^SIFUS AG, Ostermundigen, Switzerland; ^3^Departments of Neurosurgery and Neurology, Bern University Hospital, Bern, Switzerland; ^4^Department of Neurosciences, Biomedicine and Movement Sciences, University of Verona, Verona, Italy

**Keywords:** Magnetic Resonance-guided Focused Ultrasound Surgery (MRgFUS), Parkinson's disease, essential tremor, disability, quality of life

Magnetic Resonance-guided Focused Ultrasound (MRgFUS) has emerged over the past decade as one of the most promising innovations in functional neurosurgery. This Research Topic presents a comprehensive exploration of MRgFUS across 13 peer-reviewed contributions that explore MRgFUS across a broad spectrum: clinical outcomes, patient experience, neurophysiological mechanisms, targeting strategies, cognitive safety, and healthcare system integration. Together, these articles chart the maturation of MRgFUS from a novel incisionless ablative technique to a mainstream therapeutic option for movement disorders such as essential tremor (ET) and Parkinson's disease (PD).

Longitudinal studies confirm the sustained efficacy of unilateral MRgFUS thalamotomy in ET, with significant improvements in tremor severity and quality of life maintained over 3 years. Tamburin et al. report durable tremor reduction and manageable side-effect profiles in a cohort of 49 patients, reinforcing the long-term benefit of this approach. In PD, Tian et al. provide safety and efficacy analysis, while Saporito et al., in the COGNIFUS Part 2 study, demonstrate cognitive stability and mood improvements one-year post-treatment—addressing an important concern in lesional neurosurgery.

Targeting strategies are central to therapeutic success. Jameel et al., through an international survey, observe a trend toward targeting above the intercommissural plane in ventral intermediate nucleus (VIM) thalamotomies. Buch et al. further show that smaller, well-placed lesions within network-based hotspots yield greater subjective quality-of-life improvements, underscoring that “more” is not necessarily better. Complementing these findings, Blitz et al. report that the lesion size and its evolution over time as seen in MR images does not correlate with tremor control, while Bruno et al. analyze nine patients with tremor recurrence after VIM thalamotomy to extrapolate optimal targeting location. Low skull density ratio (SDR) has been and still is a limiting factor in patient eligibility due to concerns about acoustic energy transmission. Ng et al. challenge this paradigm by demonstrating that patients with an SDR < 0.40 can still benefit—albeit requiring higher sonication energy and experiencing slightly increased failure rates—prompting a re-evaluation of strict exclusion criteria.

On the neurophysiological front, Visani et al. present novel Magnetoencephalography (MEG) data indicating that MRgFUS thalamotomy induces early cortical reorganization, including reduced beta-band cortico-cortical coupling and adjusted cortico-muscular coherence. These findings offer potential early biomarkers of treatment response.

The phenomenological study by Stoycheva et al. reminds us that outcome metrics must encompass not just tremor scores but also the patient's lived experience.

Liang et al. provide a network meta-analysis comparing MRgFUS and deep brain stimulation (DBS), showing comparable efficacy in motor symptom control and quality-of-life enhancement. While DBS remains the gold standard for many, MRgFUS is increasingly validated as a viable alternative, particularly for patients ineligible for implants. Cesarano et al. further support MRgFUS's potential through a systematic review of staged bilateral treatments in both ET and PD, demonstrating encouraging safety and efficacy profiles.

Finally, Rinaldo et al. outlie a structured diagnostic-therapeutic pathway (DTP) for integrating MRgFUS into clinical workflows. Their experience with over 600 cases offers a practical and scalable model for institutions aiming to adopt or expand MRgFUS programs.

In conclusion, MRgFUS is maturing into a safe, effective, and patient-centered modality for the treatment of movement disorders. The contributions in this Research Topic highlight both technological progress and humanistic insight, reaffirming the growing role of MRgFUS in modern functional neurosurgery.

